# Isorhamnetin Inhibits Liver Fibrosis by Reducing Autophagy and Inhibiting Extracellular Matrix Formation via the TGF-*β*1/Smad3 and TGF-*β*1/p38 MAPK Pathways

**DOI:** 10.1155/2019/6175091

**Published:** 2019-07-31

**Authors:** Ning Liu, Jiao Feng, Xiya Lu, Zhilu Yao, Qing Liu, Yang Lv, Yuru Han, Jingfan Deng, Yingqun Zhou

**Affiliations:** ^1^Department of Gastroenterology, Shanghai Tenth People's Hospital, Tongji University, School of Medicine, Shanghai 200072, China; ^2^Shanghai Tenth Hospital, School of Clinical Medicine of Nanjing Medical University, Shanghai 200072, China

## Abstract

**Objective:**

Liver fibrosis is a consequence of wound-healing responses to chronic liver insult and may progress to liver cirrhosis if not controlled. This study investigated the protection against liver fibrosis by isorhamnetin.

**Methods:**

Mouse models of hepatic fibrosis were established by intraperitoneal injection of carbon tetrachloride (CCl_4_) or bile duct ligation (BDL). Isorhamnetin 10 or 30 mg/kg was administered by gavage 5 days per week for 8 weeks in the CCl_4_ model and for 2 weeks in the BDL model. Protein and mRNA expressions were assayed by western blotting, immunohistochemistry, and quantitative real-time polymerase chain reaction.

**Results:**

Isorhamnetin significantly inhibited liver fibrosis in both models, inhibiting hepatic stellate cell (HSC) activation, extracellular matrix (ECM) deposition, and autophagy. The effects were associated with downregulation of transforming growth factor *β*1 (TGF-*β*1) mediation of Smad3 and p38 mitogen-activated protein kinase (MAPK) signaling pathways.

**Conclusion:**

Isorhamnetin protected against liver fibrosis by reducing ECM formation and autophagy via inhibition of TGF-*β*1-mediated Smad3 and p38 MAPK signaling pathways.

## 1. Introduction

Liver fibrosis is associated with long-term chronic liver diseases caused by viral infection, metabolic disorders, drugs, cholestatic diseases, alcohol abuse, or immune attack [1]. Sustained liver insult leads to progressive fibrosis and ultimately to cirrhosis and hepatocellular carcinoma. Liver fibrosis represents a significant global health burden [2]. Antiviral drugs or immunosuppressive agents may prevent or attenuate liver fibrosis in a subset of cases. However, effective treatments are not available for many patients, leading to end-stage liver cirrhosis and poor prognosis [3, 4]. The lack of preventive and therapeutic interventions has created an urgent need for effective antifibrotic agents.

Liver fibrosis is characterized by overproduction of collagen type I (Col-1) and deposition of extracellular matrix (ECM) in the subendothelial space [5, 6]. Hepatic stellate cells (HSCs) are the main source of ECM. They are quiescent under physiological conditions and store hepatic retinoid and vitamin A [7]. Activation of HSCs by autocrine or paracrine stimuli promotes development of a myofibroblast-like phenotype, with loss of lipid droplets, and expression *α*-smooth muscle actin (*α*-SMA) [8]. Activated HSCs are responsive to both fibrogenic and proliferative stimuli that promote the proliferation of fibrogenic cells and the production of ECM. The activity of matrix metalloproteinase (MMP) and tissue inhibitor of matrix metalloproteinase (TIMP) enzymes, which regulate ECM formation and removal, is also disturbed in liver fibrosis [9]. The complex changes during liver fibrosis result in distortion and disruption of hepatic tissue structure and function [10]. Inhibition of HSC activation and proliferation and maintaining balanced ECM production and degradation are key potential liver fibrosis treatment targets.

Transforming growth factor *β* (TGF-*β*) is present in normal liver tissue, but increased expression occurs at all stages of progressive liver disease, including fibrosis [11]. Production of TGF-*β*1 contributes to HSC activation and excessive accumulation of ECM, thereby promoting liver fibrosis [12]. Current evidence suggests that regulating the TGF-*β*1 pathway may attenuate fibrogenesis [11, 13–16].

Autophagy helps to maintain normal liver homeostasis by removal of defective organelles and components. Autophagy initiates with the formation of autophagosomes, which are vacuoles bounded by a double membrane that fuses with lysosomes and delivers the defective components, which are then digested [17]. It is well-known that autophagy is increased with HSC activation in mouse fibrosis models and in human fibrotic liver tissue. Blocking autophagy in HSCs inhibits HSC activation and alleviates liver fibrosis [18, 19]. Thus, interventions involving autophagy in HSCs may be an attractive therapeutic target for hepatic fibrosis.

Isorhamnetin (IH) is a flavonol aglycone isolated from the plant *Hippophae rhamnoides* L. that has anti-inflammatory, antioxidant, and antitumor activity [20, 21]. IH has hepatoprotective effects by inhibiting hepatocyte autophagy and apoptosis [20]. Zheng et al. reported that IH protected against pulmonary fibrosis induced by bleomycin in mouse models by inhibiting epithelial-mesenchymal transition and endoplasmic reticulum stress [22]. Recently, Yang et al. reported that IH attenuated carbon tetrachloride- (CCl_4_-) induced liver fibrosis by downregulating TGF-*β*/Smad signaling and reducing oxidative stress [23]. However, whether IH could attenuate bile duct ligation- (BDL-) induced hepatic fibrosis is unclear, and the involvement of autophagy has not been investigated.

Based on these, this study is aimed at investigating the role of IH on CCl_4_- or BDL-induced mouse models of hepatic fibrosis, mainly focus on the involvement of the TGF-*β*1/Smad3 and TGF-*β*1/p38 MAPK pathway signaling and autophagy.

## 2. Materials and Methods

### 2.1. Reagents

IH was purchased from Sigma-Aldrich (St. Louis, MO, USA), and CCl_4_ was purchased from Sinopharm (Shanghai, China). Primers used in real-time quantitative polymerase chain reaction (qPCR) assays were from Generay (Shanghai, China). *α*-SMA (cat. no. 14395-1-AP), Col-1 (cat. no. 14695-1-AP), MMP-2 (cat. no. 10373-2-AP), TIMP1 (cat. no. 10753-1-AP), p38 (cat. no. 14064-1-AP), peroxisome proliferator-activated receptor *γ* (PPAR-*γ*; cat. no. 16643-1-AP), TGF-*β*1 (cat. no. 21898-1-AP), beclin-1 (cat. no. 11306-1-AP), and LC3 (cat. no. 14600-1-AP) antibodies were from Proteintech (Chicago, IL, USA). Phospho-p38 (p-p38; cat. no. 4511) was from Cell Signaling Technology (Danvers, MA, USA). F4/80 (cat. no. GB11027) antibody was from Servicebio (Wuhan, China). Phospho-Smad3 (p-Smad3; cat. no. ab52903) and Smad3 (cat. no. ab40854) antibodies were obtained from Abcam (Cambridge, MA, USA). Goat anti-mouse (cat. no. 926-68020) and anti-rabbit (cat. no. 926-32211) secondary antibodies were from LI-COR Biosciences (Lincoln, NE, USA).

### 2.2. Animals

Male C57 mice (22–24 g, 8 weeks old) were obtained from Shanghai Laboratory Animal Co. Ltd. (Shanghai, China) and housed under constant conditions (24°C ± 2°C, and a 12 h/12 h light/dark cycle) with a standard diet. The study was approved by the Animal Care and Use Committee of Shanghai Tongji University (Approval no. SHDSYY-2018-1474) and conducted following the National Institutes of Health Guidelines.

#### 2.2.1. Animal Experiment

A preliminary experiment was performed before the formal experiment to show the safety of IH treatment and sham operation on the liver. Mice were randomly allocated in four groups (5 animals per group): a control group that accepted no treatment, a vehicle group that was injected intraperitoneally with olive oil three times a week, a sham operation group with laparotomy only, or an IH group gavaged with 30 mg/kg IH in saline five times a week. The mice were treated for 8 weeks and sacrificed under anesthesia. Serum and hepatic tissues were obtained for biochemical assays and histopathology.

We then established the following two animal models to study the effects of IH on liver fibrosis.

Mice in the CCl_4_-induced fibrosis model were given with 10% CCl_4_ (1 mL/kg, diluted in olive oil; i.p.) three times a week for 8 weeks. IH in saline was given by gavage at either 10 or 30 mg/kg five days per week for 8 weeks. Mice were randomly allocated in four groups (8 animals per group): a vehicle control group injected intraperitoneally with olive oil, a model group intraperitoneal injection with CCl_4_, intraperitoneal injection of CCl_4_ and 10 mg/kg IH, or intraperitoneal injection of CCl_4_ and 30 mg/kg IH.

Mice in the BDL model were randomly allocated in four groups (eight animals each group): a sham control group, BDL group, BDL+10 mg/kg IH group, and BDL+30 mg/kg IH group. Surgery was performed as previously described [14]. IH was administrated 24 h after the surgery. Mice were given 10 or 30 mg/kg IH by gavage for 5 days per week for 2 weeks.

### 2.3. Biochemical Detections

Serum aspartate aminotransferase (AST) and alanine aminotransferase (ALT) were assayed with microplate detection kits (Jiancheng Bioengineering Institute, Nanjing, China) and read by spectrophotometry using an AU1000 automated chemistry analyzer (Olympus Corporation, Tokyo, Japan). Liver hydroxyproline was determined with detection kits (Biocheck, Foster City, CA, USA) according to the manufacturer's protocol. Serum TGF-*β*1 was measured using enzyme-linked immunosorbent assay (ELISA) kits (mlbio, China).

### 2.4. Histopathology

Fresh liver tissues were fixed with 4% paraformaldehyde for 2 days at room temperature, embedded in paraffin, and then sectioned at 5 *μ*m. The thin sections were stained with hematoxylin and eosin (H&E) or Masson's trichrome following standard methods [13]. The tissues were viewed under light microscopy to evaluate liver injury and extent of collagen fibers.

### 2.5. qPCR

Total RNA was isolated from frozen mouse hepatic tissues with TRIzol reagent (Tiangen Biotech, Beijing, China) according to the standard protocol. Then, 0.5 *μ*g extracted RNA was converted to cDNA through TaKaRa reverse transcription kits (Biotechnology, Dalian, China). mRNA expression was assayed with SYBR Premix EX Taq (TaKaRa Biotechnology), using a 7900HT Fast Real-Time PCR system (Applied Biosystems, Foster City, CA, USA). The sequences of primers are listed in Table 1.

### 2.6. Western Blotting

Total protein was extracted from frozen tissues and quantified by standard procedures. Proteins (80 *μ*g) were isolated by sodium dodecylsulfate-polyacrylamide gel electrophoresis, and transferred onto polyvinylidene fluoride membranes. Then, blots were blocked with 5% defatted milk powder and sequentially incubated with primary antibodies and secondary goat anti-mouse or anti-rabbit antibodies [15]. The primary antibodies and dilutions were beclin-1 (1 : 1000), LC3 (1 : 500), *α*-SMA (1 : 1000), PPAR-*γ* (1 : 500), Col-1 (1 : 500), MMP-2 (1 : 1000), TIMP1 (1 : 1000), TGF-*β*1 (1 : 500), p38 (1 : 1000), p-p38 (1 : 1000), Smad3 (1 : 500), p-Smad3 (1 : 500), and *β*-actin (1 : 1000). The secondary antibodies were at dilution of 1 : 2000. The blots were read with an Odyssey two-color infrared laser imaging system (LI-COR Biosciences).

### 2.7. Immunohistochemistry

The prepared paraffin-embedded sections were dewaxed, dehydrated, and incubated in 3% hydrogen peroxide. Antigen retrieval was performed by four cycles of heating to 100°C and cooling citrate buffer. Nonspecific protein binding sites were blocked with 5% bovine serum albumin. The sections were incubated with *α*-SMA, Col-1, beclin-1, TGF-*β*1, p-p38, p-Smad3, PPAR-*γ*, and F4/80 primary antibodies at 1 : 500 dilutions at 4°C overnight. After incubating with secondary antibody (1 : 200 dilution), antibody binding was visualized with a diaminobenzidine kit. Positive areas were observed by light microscopy.

### 2.8. Statistical Analysis

Statistical differences were evaluated using analysis of variance followed by Tukey's correction for multiple comparisons. Data were analyzed with SPSS version 20.0 (Chicago, IL, USA). A value of *p* < 0.05 was considered statistically significant.

## 3. Results

### 3.1. IH and Sham Operation Had No Harmful Effects on the Liver

**Figure 1:**
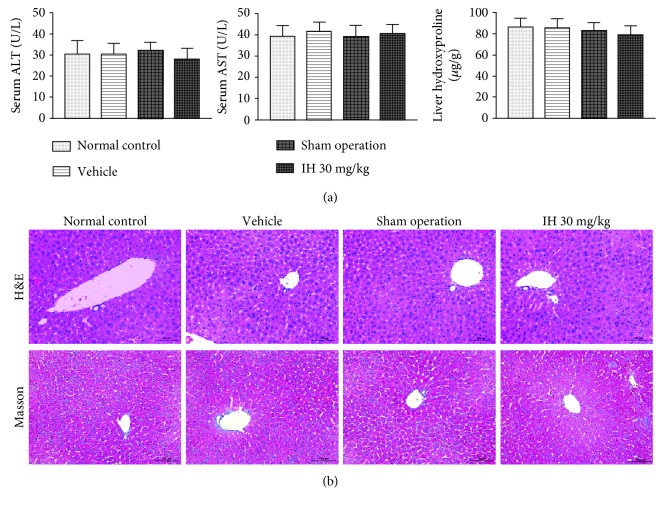
IH treatment, surgery, and the IH vehicle had no adverse effects on the liver. (a) Serum ALT and AST and liver hydroxyproline in the four study groups were not significantly different. Data are expressed as mean ± SD. (b) H&E and Masson's trichrome staining of liver tissue did not show obvious pathological changes in the four groups (original magnification, ×200; scale bar, 100 *μ*m).

Serum ALT and AST and liver hydroxyproline in normal controls, sham-operated, vehicle-treated, and IH-treated mice were not significantly different (Figure 1(a)). H&E and Masson staining did not find any obvious pathological changes in the four groups (Figure 1(b)). The results indicated that IH, vehicle, and sham operation had no harmful effects on the liver.

### 3.2. IH Attenuated Liver Fibrosis Induced by CCl_4_ and BDL in Mouse

**Figure 2:**
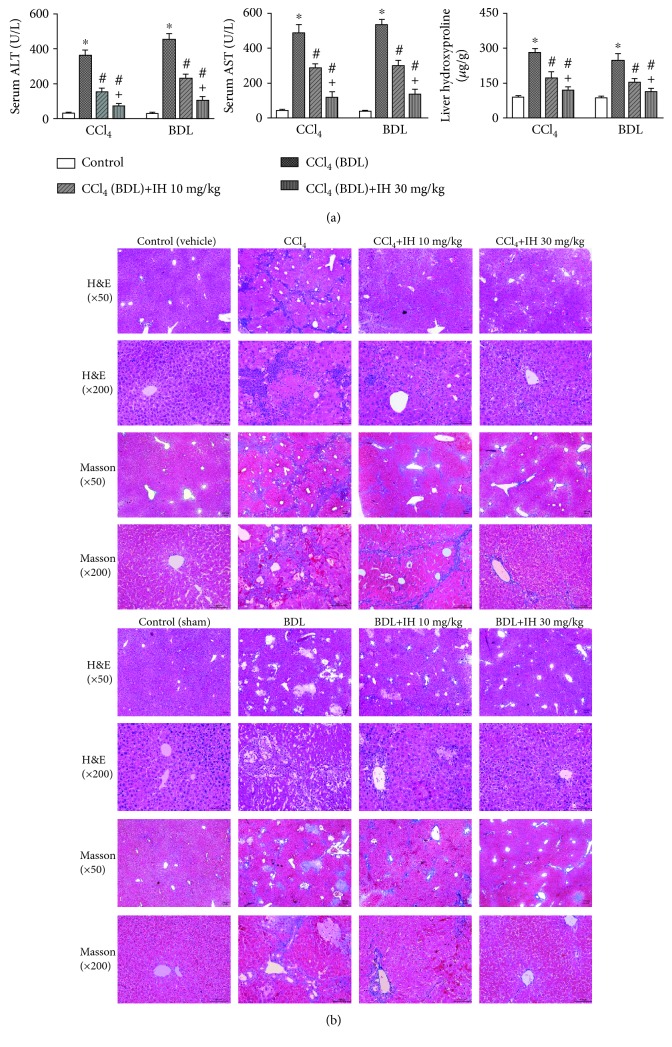
IH attenuated CCl_4_- and BDL-induced liver fibrosis in mice. (a) Effect of CCl_4_ (BDL) and IH on serum ALT and AST and liver hydroxyproline levels. Data are presented as mean ± SD (*n* = 8, ^∗^*p* < 0.05 compared to the vehicle (sham) group, ^#^*p* < 0.05 compared to the CCl_4_ (BDL) group, and ^+^*p* < 0.05 compared to the CCl_4_ (BDL)+IH 10 mg/kg group). (b) Representative images of liver sections stained with H&E and Masson's trichrome in each group (original magnification: 50x and 200x, scale bar: 100 *μ*m).

ALT and AST, two markers of liver injury, increased significantly following CCl_4_ injection and BDL surgery (Figure 2(a)). IH decreased liver enzymes in a dose-dependent manner, which indicated that IH protected the liver from chronic injury. Hydroxyproline, a constituent of liver collagen, also increased significantly in CCl_4_- and BDL-treated mice. As shown in Figure 2(a), IH dose-dependently decreased liver hydroxyproline levels. Histological evaluation (Figure 2(b)) was performed by H&E and Masson staining. H&E-stained tissue revealed that inflammatory infiltration, hepatocyte swelling and necrosis, damage of the liver lobules, and the formation of fibrous septa were prominent in both CCl_4_- and BDL-treated mice. Bile duct proliferation was also evident in mice with BDL-induced liver fibrosis. The histological changes observed in both fibrosis models were significantly attenuated by IH. The extent of the improvement increased with the IH dose. Masson staining demonstrated extensive collagen deposition in both fibrosis models. Collagen deposition was significantly reduced by IH. The results confirmed the protective effects of IH against liver fibrosis in both mouse models.

### 3.3. IH Inhibited Massive Macrophage Recruitment in the Liver

**Figure 3:**
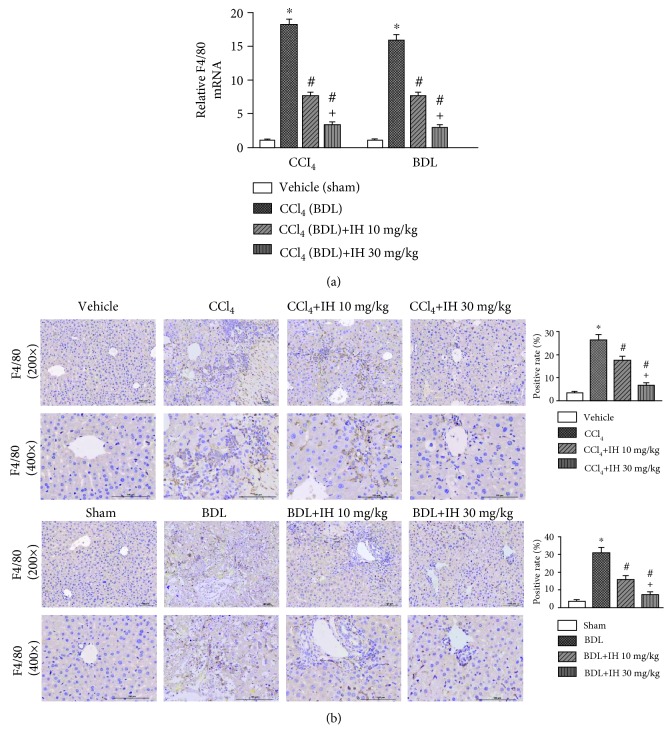
IH inhibited massive macrophage accumulation in liver tissues. (a) qPCR analysis of the F4/80 mRNA expression in liver tissues. Results are given as fold change over the control (vehicle or sham) group. (b) Representative immunostaining of F4/80 in different groups (brown indicates positive staining, original magnification: ×200 and ×400, scale bar: 100 *μ*m). Data are presented as mean ± SD (*n* = 8, ^∗^*p* < 0.05 compared to the vehicle (sham) group, ^#^*p* < 0.05 compared to the CCl_4_ (BDL) group, and ^+^*p* < 0.05 compared to the CCl_4_ (BDL)+IH 10 mg/kg group).

F4/80 is a marker of the liver macrophages (Kupffer cells). We determined the expression of F4/80 to analyze the effect of IH treatment on macrophage infiltration. The mRNA expression of F4/80 was significantly elevated in the fibrotic livers induced by CCl_4_ and BDL when compared to the normal livers determined by qPCR; however, IH markedly reduced the expression of F4/80 in liver tissues (Figure 3(a)). Immunohistochemistry of F4/80 showed that spindle-shaped F4/80-positive cells increased significantly in the hepatic fibrosis groups compared with the control groups. However, the elevation was obviously inhibited by IH treatment (Figure 3(b)). The results indicated that IH could inhibit chronic CCl_4_- and cholestasis-induced massive macrophage recruitment in liver tissues.

### 3.4. IH Inhibited HSC Activation and ECM Formation

**Figure 4:**
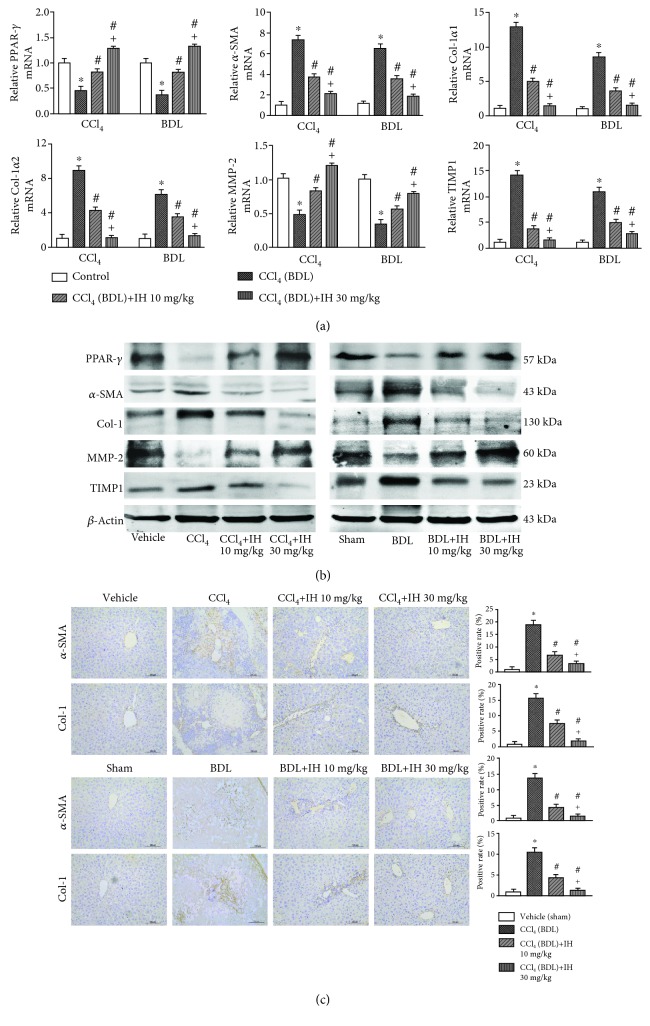
IH attenuated ECM accumulation in livers. (a) qPCR was used to determine the mRNA expression of PPAR-*γ*, *α*-SMA, Col-1*α*1, Col-1*α*2, MMP-2, and TIMP1 in liver tissues. Results are given as fold change over the control (vehicle or sham) group. (b) Western blotting was used to detect the protein expression of PPAR-*γ*, *α*-SMA, Col-1, MMP-2, and TIMP1 in liver tissues in mice of each group. (c) Immunohistochemical staining was performed to analyze the expression of *α*-SMA and Col-1 in liver sections (brown indicates positive staining, original magnification: ×200, scale bar: 100 *μ*m). Data are presented as mean ± SD (*n* = 8, ^∗^*p* < 0.05 compared to the vehicle (sham) group, ^#^*p* < 0.05 compared to the CCl_4_ (BDL) group, and ^+^*p* < 0.05 compared to the CCl_4_ (BDL)+IH 10 mg/kg group).

*α*-SMA and PPAR-*γ* are considered as markers of HSC activation and quiescence, respectively [24–26]. mRNA and protein levels of *α*-SMA were significantly upregulated, whereas the PPAR-*γ* expression was markedly downregulated in mice exposed to chronic CCl_4_ and BDL compared with controls. IH dose-dependently reduced the *α*-SMA expression and increased the PPAR-*γ* expression in liver tissues. Collagen (especially types I and III) is the main component of ECM in liver tissues. The qPCR, western blotting, and immunohistochemistry results showed that the Col-1 expression in the liver was obviously elevated in both fibrosis model mice compared with controls, whereas IH significantly reduced the collagen expression in liver tissues (Figures 4(a)–4(c)). MMP-2 has been shown to be involved in suppressing the collagen expression, and TIMP1 overexpression has been associated with inhibiting ECM clearance [27, 28]. As shown in Figures 4(a) and 4(b), the MMP-2 expression was significantly decreased, while the expression of TIMP1, an MMP inhibitor, was increased in both fibrosis models. As shown by qPCR and western blotting, both mRNA and protein expressions were affected in the fibrosis models. The results are consistent with the activation of HSC and overproduction and impaired degradation of ECM in the fibrosis models. IH decreased the expression of TIMP1 and increased the MMP-2 expression at both mRNA and protein levels in a dose-dependent manner (Figures 4(a)–4(c)). The results showed that IH inhibited HSC activation and maintained the balance of ECM production and degradation in both fibrosis models.

### 3.5. IH Reduced Autophagy in Both Liver Fibrosis Models

**Figure 5:**
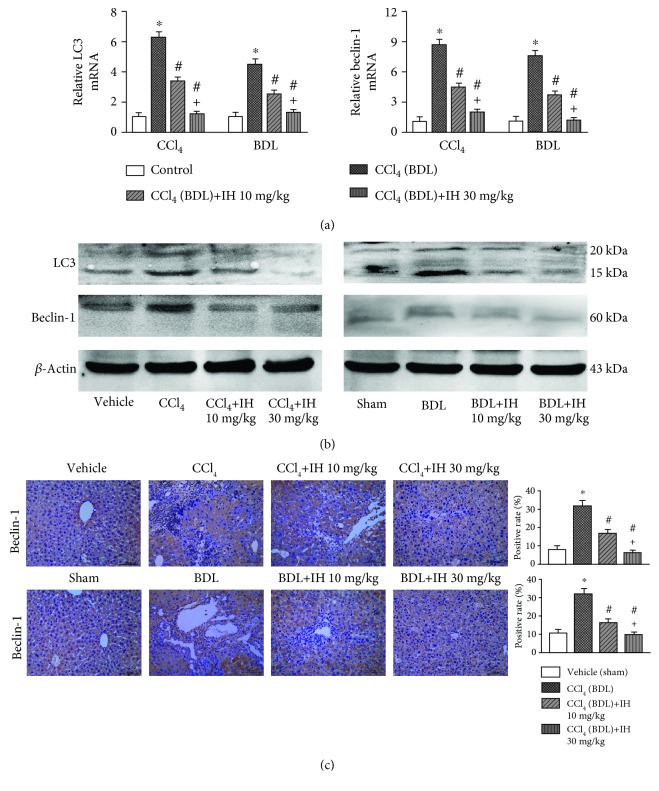
IH inhibited autophagy in fibrotic hepatic tissues. (a) The mRNA expression of LC3 and beclin-1 in liver tissues was analyzed by qPCR. Results are given as fold change over the control (vehicle or sham) group. (b) Protein levels of LC3 and beclin-1 were analyzed by western blotting. (c) Beclin-1 protein expression was analyzed using immunohistochemistry (brown indicates positive staining, original magnification: ×200, scale bar: 100 *μ*m). Data are presented as mean ± SD (*n* = 8, ^∗^*p* < 0.05 compared to the vehicle (sham) group, ^#^*p* < 0.05 compared to the CCl_4_ (BDL) group, and ^+^*p* < 0.05 compared to the CCl_4_ (BDL)+IH 10 mg/kg group).

Beclin-1 and LC3 expressions are associated with autophagosome formation and considered autophagy markers. mRNA and protein levels of beclin-1 and LC3 were significantly elevated in both fibrosis models compared with control mice (Figures 5(a) and 5(b)); however, IH prevented their increase in a dose-dependent way. The results of beclin-1 immunohistochemical staining were consistent with the results of western blotting (Figure 5(c)). The results indicated that IH inhibited autophagy in both liver fibrosis mouse models.

### 3.6. IH Downregulated TGF-*β*1-Activated Smad3 and p38 MAPK Signaling Pathways

**Figure 6:**
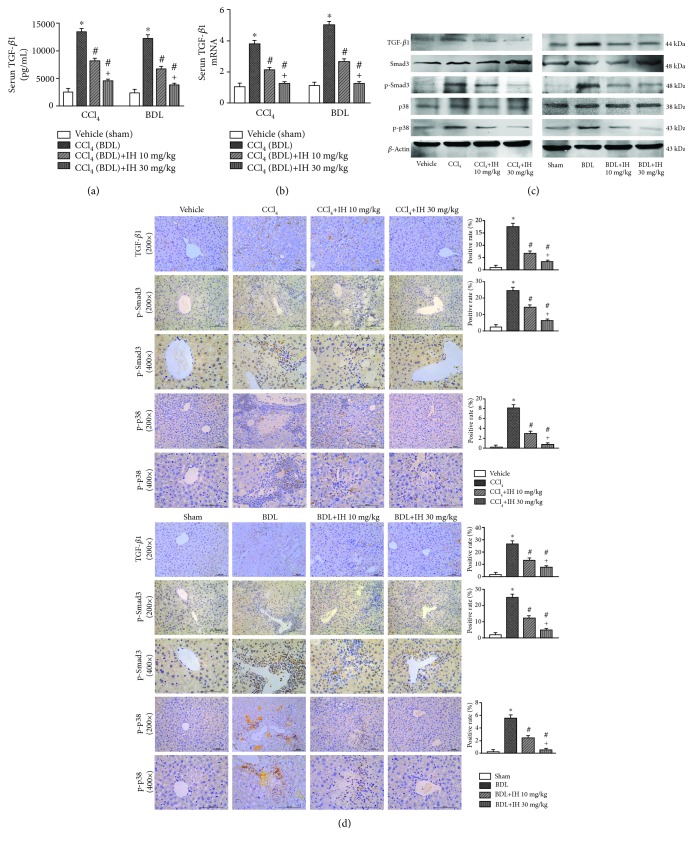
IH regulated the TGF-*β*1-mediated Smad3 and p38 MAPK signaling pathways. (a) The serum level of TGF-*β*1 was detected by ELISA. (b) The mRNA expression of TGF-*β*1 in the liver was analyzed by qPCR. Results are given as fold change over the control (vehicle or sham) group. (c) The protein expression of TGF-*β*1, p-Smad3, and p-p38 MAPK was analyzed by western blotting. (d) The protein expression of TGF-*β*1, p-Smad3, and p-p38 MAPK in liver sections was analyzed using immunohistochemical staining (brown indicates positive staining, original magnification: ×200 and ×400, scale bar: 100 *μ*m). Data are presented as mean ± SD (*n* = 8, ^∗^*p* < 0.05 compared to the vehicle (sham) group, ^#^*p* < 0.05 compared to the CCl_4_ (BDL) group, and ^+^*p* < 0.05 compared to the CCl_4_ (BDL)+IH 10 mg/kg group).

The expression of TGF-*β*1, the most potent fibrogenic cytokine in the liver, was prominently increased in both fibrosis models, but IH treatment significantly decreased both mRNA and protein levels of TGF-*β*1 in liver tissues (Figures 6(b)–6(d)). The changes of TGF-*β*1 in serum were consistent with those in liver tissues (Figure 6(a)). We then focused on p-Smad3 and p-p38 MAPK, which are the downstream signaling molecules of TGF-*β*1. As shown by western blot assays and immunohistochemistry (Figures 6(c) and 6(d)), the expression of p-Smad3 and p-p38 MAPK proteins was upregulated in liver fibrosis tissues compared with controls, while both were dose-dependently attenuated by IH. The results demonstrated that IH improved liver fibrosis through downregulating TGF-*β*1 mediation of Smad3 and p38 MAPK signaling.

## 4. Discussion

Hepatic fibrosis, or liver scarring, is a response to liver injury. Sustained insult may lead to progression of liver fibrosis that produces serious, irreversible liver disease, cirrhosis, and hepatic carcinoma [1, 5, 6]. Therapeutic strategies for liver fibrosis are still limited. Studies showed that blocking the TGF-*β*1 pathway could be a potential strategy for liver fibrosis [13–16]. T*β*R antagonists could effectively inhibit the TGF-*β* pathway and showed the antifibrotic potency both in vitro and in vivo [29, 30]. However, the on-target toxicity of T*β*R antagonists, especially cardiac injuries, should be highly concerned. The concentration must be carefully controlled to reduce the toxicity, which limited the development and application of T*β*R antagonists [31–33]. Therefore, discovering an effective and safe therapeutic agent for liver fibrosis is urgently needed. Our previous study showed that IH treatment is hepatoprotective [20]. Recent studies also showed the protective role of IH in cardiovascular diseases [34], lung injury [35], and renal damage [36]. These studies demonstrated that IH treatment is safe and protective for multiple organ function in animal models. Remarkably, IH has previously been reported to show antifibrosis activity in lung and cardiac muscle [22, 37]. IH was also found to reduce the extent of CCl_4_-induced hepatic fibrosis by reducing oxidative stress [23]. Therefore, IH might be a promising candidate for hepatic fibrosis treatment.

In this study, we investigated the antifibrotic effects of IH in both CCl_4_- and BDL-induced mouse models of liver fibrosis. Intraperitoneal injection of CCl_4_ is a reliable method for the establishment of stable fibrotic models. The pathological appearance is similar to that of human hepatic fibrosis caused by hepatitis B infection [38]. BDL-induced fibrosis is similar to injury caused by cholestatic obstruction [39]. Our results showed that IH at both 10 and 30 mg/kg effectively protected hepatic function and improved liver pathology changes in both fibrosis models. The therapeutic mechanism of IH in liver fibrosis involves downregulating the TGF-*β*1/Smad3 and TGF-*β*1/p38 MAPK pathways. Therefore, the present study indicated that IH could be an attractive agent for the clinical therapy of liver fibrosis.

ALT and AST are two liver enzymes that reflect hepatocyte integrity. Liver enzymes leak into the bloodstream by liver injury and increase their levels in serum, which reflects hepatocyte injury [40]. The elevated ALT and AST levels were recovered following IH treatment in both fibrotic models, which suggested that IH protected hepatocytes from chronic injury. In parallel, IH treatment alleviated inflammation infiltration in liver sections. Our previous study also showed that IH inhibited hepatocyte apoptosis and autophagy induced by inflammation, thereby improving liver function [20]. It indicated that the hepatoprotective role of IH could be attributed to the anti-inflammation effect [41, 42].

The importance of HSC activation in hepatic fibrosis is well known. Chronic damage of hepatic tissue leads to activation of resting HSCs and transdifferentiation into myofibroblasts. The myofibroblasts express *α*-SMA, lose their lipid droplets, and contribute to the formation of ECM [8]. The progressive accumulation of ECM in the Disse space produces mechanical irritation that continues to drive HSC activation [24], and the binding and accumulation of growth factors within the ECM further enhance HSC proliferation [24, 43]. Therefore, decreased matrix deposition may attenuate liver fibrosis. ECM production is modulated by TIMPs and MMPs. MMPs degrade ECM proteins, while TIMPs inhibit the proteolytic activity of MMPs. The balance between MMP and TIMP activity is disturbed in liver fibrosis [28, 44]. Shifting the MMP-TIMP balance facilitates the deposition of ECM. In this study, changes in *α*-SMA and Col-1 expressions revealed that HSC activation and ECM accumulation were reduced in liver tissues treated with IH. We also examined the expression of PPAR-*γ*, a quiescent HSC marker, in liver tissues. Studies have shown that PPAR-*γ* is involved in maintaining the quiescent HSC phenotype and acts as a negative regulator in HSC transdifferentiation [25, 26]. The induction of PPAR-*γ* by IH reflects the alleviation of liver fibrosis and also plays a great role in maintaining quiescence of HSCs [45, 46]. Additionally, the study confirmed, as would be expected, that the balanced expression of MMP-2 and TIMP1 was changed in both fibrosis models, but it was recovered by IH treatment. The results indicate that IH attenuated liver fibrosis by inhibiting HSC activation and regulating ECM balance.

Immune cells, especially liver macrophages (Kupffer cells), also play a pivotal role in hepatic fibrosis. In response to liver injury, massive Kupffer cells recruit to the liver, and produce inflammatory cytokines (such as TGF-*β*1), which perpetuate inflammation and mediate HSC activation [47, 48]. Our result indicated that IH could effectively inhibit inflammation and regulate Kupffer cell recruitment in the chronic damaged liver, which contributed to the alleviation of liver fibrosis caused by chronic CCl_4_ and cholestasis exposure [48].

TGF-*β*1 is a fibrogenic cytokine secreted by both liver macrophages (Kupffer cells) and activated HSCs [11], and it promotes transdifferentiation and proliferation of HSCs. It targets the *α*1 and *α*2 procollagen type I genes and TIMP-1 and TIMP-2 expressions [11, 49–52]. The TGF-*β*1 expression mediates intracellular signaling through the Smad and p38 MAPK pathways [53]. Binding of TGF-*β* to the TGF-*β* receptor (T*β*R) type II transphosphorylates T*β*R type I, which subsequently phosphorylates downstream pathways of Smad [11, 54]. The phosphorylated Smad protein then combines with Smad4, and the complex translocates into the nucleus where it regulates the transcription of genes coding collagens, *α*-SMA, and TIMP1 [12, 55, 56]. Suppression of Smad3 in HSCs is known to inhibit the Col-1 expression and reduce liver fibrosis [12, 57, 58]. In addition, other signal transducers such as the p38 MAPK pathway can also be medicated by activation of T*β*R. Previous studies have found that p38 MAPK signaling independently, and additively along with Smad, regulates collagen transcription [59–61]. p38 MAPK signaling also increases the stability of the Col-1 mRNA expression [61]. The evidence supports fibrogenic activity by Smad3 and p38 MAPK in TGF-*β*1-induced hepatic fibrosis. In this study, we explored the effect of IH on TGF-*β*1/Smad3 and TGF-*β*1/p38 MAPK pathways. The results found that IH blocked the increase of the TGF-*β*1 expression during liver injury. Accordingly, the downstream molecules, p-Smad3 and p-p38, were downregulated with IH treatment. Therefore, IH treatment efficiently blocked the TGF-*β*1 pathway, which was involved in the antifibrosis activity of IH.

Autophagy maintains liver homeostasis by degrading and recycling aggregated proteins and damaged organelles [17], but inappropriate activation of autophagy has been associated with various diseases, including liver fibrosis [17, 62]. Autophagy provides energy through digestion of intracellular lipids to support HSC activation and promote fibrosis [18, 63]. Knockout of the autophagy-related Atg5 gene, or treatment with the autophagy inhibitor 3-methyladenine, reduces HSC activation [19, 64]. Treating HSCs with 3-methyladenine also inhibits HSC proliferation [62]. Autophagy thus appears to be required for both HSC activation and proliferation and may be a novel target for the therapy of liver fibrosis. Our previous work also shows that inhibiting autophagy plays an important role in attenuating liver fibrosis [13–16]. Lu et al. reported that IH protected the liver against acute concanavalin A-induced injury by inhibiting autophagy [20]. In our study, we found that IH inhibited autophagy in both liver fibrosis models. It is reported that TGF-*β*1 can upregulate autophagy [65] and inhibiting TGF-*β*1/Smad3 signaling can suppress autophagy [13, 66]. Inhibition of the TGF-*β*1/Smad3 pathway by IH was confirmed in this study. Therefore, we concluded that IH reduced autophagy and alleviated liver fibrosis by downregulating the TGF-*β*1/Smad3 signaling pathway.

**Figure 7:**
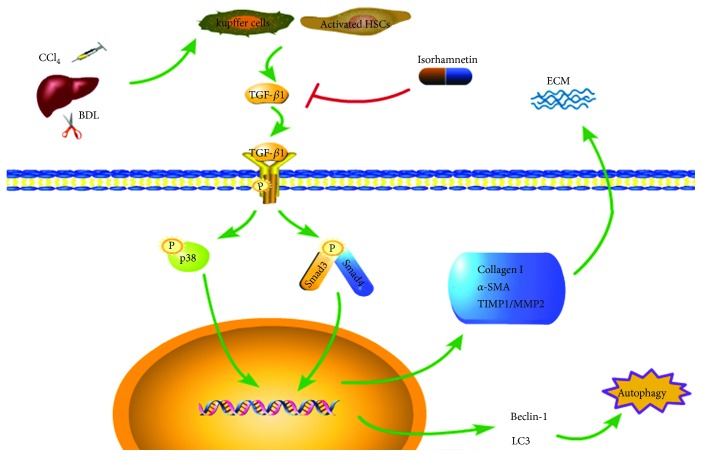
Mechanisms of IH protection against liver fibrosis. IH inhibited production of TGF-*β*1, thereby downregulating the TGF-*β*1/Smad3 and TGF-*β*1/p38 MAPK pathways. Decreased TGF-*β*1 reduced the activation of downstream Smad3 and p38 MAPK signaling, which inhibited the transcription of *α*-SMA, collagen, and TIMP1 genes to suppress ECM production. Autophagy is reduced because of the inhibition of the TGF-*β*1/Smad3 signaling pathway. Complex, IH attenuated liver fibrosis in mice by inhibiting autophagy and ECM formation. Downregulation of the TGF-*β*1-mediated Smad3 and p38 MAPK pathways was involved.

In summary, IH alleviated liver fibrosis induced by CCl_4_ and BDL in mice (Figure 7). It inhibited the production of TGF-*β*1 secreted by Kupffer cells and activated HSCs. A lack of TGF-*β*1 downregulated downstream signaling by the Smad3 and p38 MAPK signaling pathways, contributing to the inhibition of HSC activation and ECM production. Autophagy was inhibited by downregulation of the TGF-*β*1/Smad3 signaling pathway, and the resulting energy deficit decreased HSC activation.

The study demonstrates the potential protective role of IH against both noncholestatic and cholestatic fibrosis. This is the first study to show that inhibiting autophagy in HSCs is involved in the antifibrotic mechanism of IH. It also highlights the key role of regulating autophagy and TGF-*β*1 pathways in the treatment of hepatic fibrosis. However, other mechanisms of action and the safety of IH for clinical applications remain to be investigated.

## 5. Conclusions

The study confirmed the protective effects of IH against liver fibrosis in mice. The mechanism of the antifibrotic effects of IH involves inhibition of TGF-*β*1-mediated Smad3 and p38 MAPK signaling pathways, thereby reducing autophagy and ECM formation.

## Figures and Tables

**Table 1:** Nucleotide sequences of the primers used in the qPCR assays.

Gene	Amplicon size	Primer sequence	Position
*β*-Actin	154	F: 5′-GGCTGTATTCCCCTCCATCG-3′	84-103
		R: 5′-CCAGTTGGTAACAATGCCATGT-3′	237-216
*α*-SMA	104	F: 5′-CCCAGACATCAGGGAGTAATGG-3′	118-139
		R: 5′-TCTATCGGATACTTCAGCGTCA-3′	221-200
F4/80	127	F: 5′-CTGCACCTGTAAACGAGGCTT-3′	168-188
		R: 5′-GCAGACTGAGTTAGGACCACAA-3′	294-273
Col-1*α*1	91	F: 5′-GCTCCTCTTAGGGGCCACT-3′	30-48
		R: 5′-ATTGGGGACCCTTAGGCCAT-3′	120-101
Col-1*α*2	222	F: 5′-TCGTGCCTAGCAACATGCC-3′	49-67
		R: 5′-TTTGTCAGAATACTGAGCAGCAA-3′	270-248
TIMP1	108	F: 5′-CGAGACCACCTTATACCAGCG-3′	256-276
		R: 5′-ATGACTGGGGTGTAGGCGTA-3′	335-317
MMP-2	75	F: 5′-GGACAAGTGGTCCGCGTAAA-3′	652-671
		R: 5′-CCGACCGTTGAACAGGAAGG-3′	726-707
Beclin-1	149	F: 5′-ATGGAGGGGTCTAAGGCGTC-3′	1-20
		R: 5′-TGGGCTGTGGTAAGTAATGGA-3′	149-129
LC3	322	F: 5′-GACCGCTGTAAGGAGGTGC-3′	43-61
		R: 5′-AGAAGCCGAAGGTTTCTTGGG-3′	364-344
TGF-*β*1	91	F: 5′-CCACCTGCAAGACCATCGAC-3′	92-111
		R: 5′-CTGGCGAGCCTTAGTTTGGAC-3′	182-162
PPAR-*γ*	121	F: 5′-GGAAGACCACTCGCATTCCTT-3′	69-89
		R: 5′-GTAATCAGCAACCATTGGGTCA-3′	189-168

## Data Availability

The data used to support the findings of the current study are available from the corresponding author on reasonable request.
